# Predicting diabetic retinopathy and identifying interpretable biomedical features using machine learning algorithms

**DOI:** 10.1186/s12859-018-2277-0

**Published:** 2018-08-13

**Authors:** Hsin-Yi Tsao, Pei-Ying Chan, Emily Chia-Yu Su

**Affiliations:** 1 Graduate Institute of Biomedical Informatics, College of Medical Science and Technology, Taipei Medical University, Taipei, 106 Taiwan; 2 Division of Endocrinology and Metabolism, Department of Internal Medicine, Sijhih Cathay General Hospital, New Taipei City, 221 Taiwan; 3 Department of Occupational Therapy and Healthy Aging Center, Chang Gung University, Taoyuan, 333 Taiwan; 4 Department of Psychiatry, Linkou Chang Gung Memorial Hospital, Taoyuan, 333 Taiwan; 5 Clinical Big Data Research Center, Taipei Medical University Hospital, Taipei, Taiwan

**Keywords:** Diabetic retinopathy, Clinical decision support, Machine learning, Risk factors

## Abstract

**Background:**

The risk factors of diabetic retinopathy (DR) were investigated extensively in the past studies, but it remains unknown which risk factors were more associated with the DR than others. If we can detect the DR related risk factors more accurately, we can then exercise early prevention strategies for diabetic retinopathy in the most high-risk population. The purpose of this study is to build a prediction model for the DR in type 2 diabetes mellitus using data mining techniques including the support vector machines, decision trees, artificial neural networks, and logistic regressions.

**Results:**

Experimental results demonstrated that prediction performance by support vector machines performed better than the other machine learning algorithms and achieved 79.5% and 0.839 in accuracy and area under the receiver operating characteristic curve using percentage split (i.e., data set divided into 80% as trainning and 20% as test), respectively. Evaluated by three-way data split scheme (i.e., data set divided into 60% as training, 20% as validation, and 20% as independent test), our method obtained slightly lower performance compared to percentage split, which suggested that three-way data split is a better way to evaluate the real performance and prevent overestimation. Moreover, we incorporated approaches proposed in previous studies to evaluate our data set and our prediction performance outperformed the other previous studies in most evaluation measures. This lends support to our assumption that appropriate machine learning algorithms combined with discriminative clinical features can effectively detect diabetic retinopathy.

**Conclusions:**

Our method identifies use of insulin and duration of diabetes as novel interpretable features to assist with clinical decisions in identifying the high-risk populations for diabetic retinopathy. If duration of DM increases by 1 year, the odds ratio to have DMR is increased by 9.3%. The odds ratio to have DR is increased by 3.561 times for patients who use insulin compared to patients who do not use insulin. Our results can be used to facilitate development of clinical decision support systems for clinical practice in the future.

## Background

Diabetic retinopathy (DR) is the most common cause of newly diagnosed blindness every year, especially in working-age population. Retrospective reviews of the United Kingdom Prospective Diabetes Study (UKPDS) and the Diabetes Control and Complications Trial (DCCT) on type 2 and type 1 diabetes mellitus, respectively, both suggested that intensive blood glycemic control can effectively reduce the risk of microvascular complications including diabetic retinopathy. Past studies mostly focused on controlling one major risk factor which is glucose level, whereas few studies have focused on investigating different risk factors of DR [1]. Diabetic retinopathy is microvascular complication of diabetes mellitus. It dependents on history of diabetes-related complications [2]. Diabetic retinopathy is a highly specific vascular complication of both type 1 and type 2 diabetes, with prevalence strongly related to the duration of diabetes [2]. Diabetic retinopathy consists of non-proliferative diabetic retinopathy (NPDR) and proliferative diabetic retinopathy (PDR). NPDR is also known as background diabetic retinopathy (BDR). NPDR is early stage of DR, and PDR is late stage of DR. In NPDR status, microaneurysm, hemorrhage, hard exudates, cotton wool spot, intraretinal microvascular abnormalities, and venous beading are usual characters. In PDR stage, there are disc neovascularization, vitreous hemorrhage, and fibrous scarring. Macular edema is deposition of hard exudates near macula. Diabetic retinopathy is the most frequent cause of new cases of blindness among adults aged 20–74 years [2]. Approximately 21% of the newly diagnosed patients with type 2 diabetes (T2D) were also found to have co-morbid condition of DR, whereas 60% of the patients with a chronic history of 20 years of T2D were diagnosed with diabetic retinopathy [3]. About 20–40% of patients in T2D had diabetic retinopathy and 8% of patients in T2D had sight-threatening diabetic retinopathy (STDR) in United States [4]. In 2009, Prevalence of the diabetic retinopathy and poor vision/blindness in Taiwanese patients with T2D were 8.91 and 0.62%, respectively [5].

### Treatment and screening of diabetic retinopathy

There are several treatments for DR. First, it is crucial to promptly refer patients with any level of macular edema, severe NPDR, or any PDR to an ophthalmologist who is knowledgeable and experienced in the management and treatment of diabetic retinopathy [2]. In addition, laser photocoagulation should be considered for eyes with clinically significant macular edema, particularly when the center of the macula is involved or imminently threatened [6]. Anti-vascular endothelial growth factor (anti-VEGF) therapy is also indicated for diabetic macular edema [2].

To minimize the odds of visual loss or new onset of blindness of diabetic retinopathy, current guidelines of Taiwan diabetic association suggests that the screening of fundus examination in patients with T2D needs to be performed annually, and performed more frequently in patients with diabetic retinopathy. It is noted that the screening rate was low, for there was only 28.9% of patients with T2D had eye fundus examination in Taiwan in 2009 [7]. One possible explanation for the low screening rate may be that patients with T2D do not care about retinopathy when they have normal vision with NPDR. But once they developed PDR with vitreous hemorrhage, they lost the vision suddenly. Before vision loss, photocoagulation can avoid hemorrhage of PDR and reduced vision loss. Although education of complication of T2D in our care unit is regular routine education for patients with T2D, lack of insight seems to be the major cause.

### Risk factors of diabetic retinopathy

One of the major risk factors examined in a pooled analysis from population-based studies around the world was the long duration of diabetes [8]. Other risk factors identified in this study were high level hemoglobin A1C (HbA1C) and high blood pressure [8]. According to the UKPDS, the incidence of diabetic retinopathy is closely associated with the increasing duration of T2D, and lower level of HbA1c can decrease the risk of suffering from DR in these patients [9]. However, it was observed in clinical practice that some patients with long-term controlled HbA1c levels still have risks suffering from diabetic retinopathy in T2D [10]. This suggests that the HbA1C level is not the only major risk factor, and other factors such as hypertension, high blood glucose, and duration of diabetes may have potentially played partial roles in the development of diabetic retinopathy in T2D. There were known risk factors of diabetic retinopathy such as long duration of diabetes, poor glycemic control, hypertension, and hyperlipidemia. In summary, the most common risk factor discovered by every piece of empirical evidence is duration of diabetes. Poor glycemic control leads high fasting glucose level, high, postprandial glucose, and high HbA1C. All three above mentioned parameters inform different aspects of diabetes and should all be considered.

### Prediction of diabetic retinopathy using data mining approaches

Several studies [11–16] have been developed to predict diabetic retinopathy. A cross-sectional study on patients with T2D used routinely collected data at outpatient clinics of the Isfahan Endocrinology and Metabolism Research Center (IEMRC), Iran [11]. This study applied receiver operating characteristic (ROC) curves to identify the optimum value of diabetic patients for determining DR; sensitivity and specificity for predicting DR were calculated for different cuts of score. This study demonstrated the results of using logistic regression models with DR as dependent variable. Area under the ROC curve (AUC) was 0.704, and also showed sensitivity (60%) and specificity (69%) of a risk score ≥ 52.5 for DR.

Another study discussed individual risk assessment and information technology to screen the frequency of diabetic retinopathy [12]. This study used a mathematical algorithm created using epidemiological data on risk factors for diabetic retinopathy, through a website, http://risk.is/, in which the algorithm receives clinical data, including type and duration of diabetes, HbA1c or mean blood glucose, blood pressure and the presence and grade of retinopathy. The AUC was 0.76, and this number indicates the model predicts the probability of a patient who develops sight-threatening retinopathy (STR) 76% more correct than who does not develop STR.

A study by Semeraro et al. predicted risk of diabetic retinopathy using the c-statistic, survival receiver operating characteristic, and the Gonen and Heller concordance probability estimate (CPE) for the Cox proportional hazard model [13]. For the internal validation, the C-index reached a value of 0.746; the Gonen–Heller CPE for the Cox proportional hazard method was 0.683, meaning a good level of concordance between observed occurrence of DR and that predicted by the model. For the external validation, the values for C-index and CPE were 0.767 and 0.697, respectively. The AUC for 1-year survival from retinopathy was 0.825. There was no statistical difference between the C-index of that calculated in the train data set versus that calculated on the test data set (*p* = 0.137). Then, the study use the classification and regression tree (CART) analysis or the random forest analysis for the train data set to verify how the results were consistent with these different approaches.

### Challenges of diabetic retinopathy prediction and specific aims of this study

The risk factors of DR were investigated extensively in the past studies, but it remains unknown which risk factors were more associated with the DR than others. If we can detect the DR related risk factors more accurately, we can then exercise early prevention strategies for DR in the most high-risk population. Therefore, the purpose of this study is to build a predicting model for the DR in type 2 DM using the data mining techniques including decision trees, support vector machines, artificial neural networks, and logistic regressions. It is anticipated that the results of this study will assist with clinical decisions in identifying the high-risk populations for DR.

Development a model to analyze the characteristics of the patients in order to identify the high risk population for DR is essential. There is a limited amount of research in clinical applications using data mining techniques in the current literature. The aim of the study is to identify high risk factors for DR in patients with diabetes by building a predictive model to inform the high-risk groups for eye fundus examination, and help decrease the frequency of usage in the low-risk groups to enhance cost-effectiveness in the health insurance system. This study will identify related biomedical features from patients and build predictive models to support decision making in order to reach the goal of identifying high-DR-risk population. The data mining techniques can be used to predict possible outcomes to support decision making processes. By combining the level of correlations of patient characteristics analyzed by different machine learning algorithms, we can study the risk factors of DR.

The knowledge of medicine has not been fully discovered due to high complexity of human diseases and tremendous amounts of unraveled biomedical information. Thus, using computational approaches to investigate crucial clinical features and develop clinical decision support systems is highly desirable. We attempt to use several machine learning algorithms, including decision trees (DT), support vector machines (SVM), logistic regression (LR), and artificial neural networks (ANN), to predict DR. A decision tree model is applied to assist with clinical decision making via the collection of related features of specific disease and a logistic regression model is used to identify discriminative features for diabetic retinopathy.

## Results

### Data collection and feature extraction

We used the information of a group of regular outpatients lasting for at least one year (2012/1~ 2012/12). The data was extracted for one season selected randomly from the “DM shared care” database in a private hospital in northern Taiwan. Those with fundus examination were further selected by the SAS Enterprise Guide version 5.1. A total of 536 selected patients’ data were further divided into 2 classes: normal (*n* = 430), diabetic retinopathy (DR) (*n* = 106), and DR included background DR and proliferative DR. These data further served as the database for data mining analysis in our study. The imbalanced numbers of subjects between the two groups may potentially leads to a biased result favoring the bigger group. In order to solve the problem, 106 subjects were randomly drawn out of the 430 subjects in the normal group to compare with the DR group.

There were 10 predicting features identified for this study: systolic blood pressure (SBP), diastolic blood pressure (DPB), body mass index (BMI), age, gender, duration of disease, family history of diabetes, self-monitoring blood glucose (SMBG), exercise, and insulin treatment. Categorical data are gender (male = 1, female = 2), family history, SMBG, exercise, and insulin treatment (0 = no, 1 = yes). The remaining predicting features were continuous data.

### Descriptive statistical analysis

We applied chi-squared test and *t*-test to analyze the statistical significance of categorical variables and numerical variables, respectively. Table 1 shows the counts and percentages of DM and normal groups for each categorical variable, while Table 2 illustrates that statistical analysis (i.e., minimum, maximum, mean, and standard deviation) of DM and normal groups for each numerical variable. In the categorical and numeric variables of features between DR and normal, such as exercise, family history, SMBG, and gender were not significant. But insulin, hypertension, BMI, age, and duration of diabetes were significant difference between DR and normal. This demonstrated that our preliminary statistical analysis can identify discriminative risk factors that correspond well with biomedical insights. Among the variables with statistical significance, it is interesting to observe that use of insulin and duration of diabetic obtained *p*-values less than 0.0001. This also suggests that the variables representing clinical care of diabetic patients could serve as important indicators for diabetic retinopathy prediction.

**Table 1:** Statistical analysis of categorical variables

	Value	Retinopathy				*p*-value
		DM		Normal		
		Count	Percentage (%)	Count	Percentage (%)	
Exercise	Y	60	56.60	39	36.79	0.3266
	N	46	43.40	67	63.21	
Family history	Y	60	56.60	37	34.91	0.2054
	N	46	43.40	69	65.09	
Insulin	Y	40	37.74	10	9.43	< 0.0001^*^
	N	66	62.26	96	90.57	
SMBG	Y	67	63.21	61	57.55	0.3995
	N	39	36.79	45	42.45	
Gender	F	51	48.11	62	58.49	0.1300
	M	55	51.89	44	41.51	

Counts and percentages of categorical variables between DR and normal patients are calculated

^*^Variables with *p*-value < 0.05 are highlighted

**Table 2:** Statistical analysis of numerical variables

	Retinopathy								*p*-value
	DM				Normal				
	Min	Max	Mean	StdDev	Min	Max	Mean	StdDev	
SBP	96	223	137.89	18.76	101	196	132.25	15.72	0.0188^*^
DBP	55	112	78.75	10.39	28	101	75.73	11.24	0.0435^*^
BMI	17.6	38.1	25.99	3.75	19.6	49.3	27.35	5.08	0.0278^*^
Age	35	88	61.50	10.77	19	84	57.36	12.92	0.0120^*^
≧65	65	88	71.63	5.25	65	84	72.25	4.89	0.6054^*^
40–64	43	64	55.51	5.82	41	64	53.88	5.88	0.1217^*^
< 40	35	39	36.75	2.06	19	38	28.75	6.31	0.0363^*^
Duration	1	36	12.88	7.93	1	23	7.50	5.18	< 0.001^*^

Minimum (Min), maximum (Max), mean, and standard deviation (StdDev) of numerical variables between DR and normal patients are calculated

^*^Variables with p-value < 0.05 are highlighted

### Prediction performance evaluated by percentage split

**Fig. 1:**
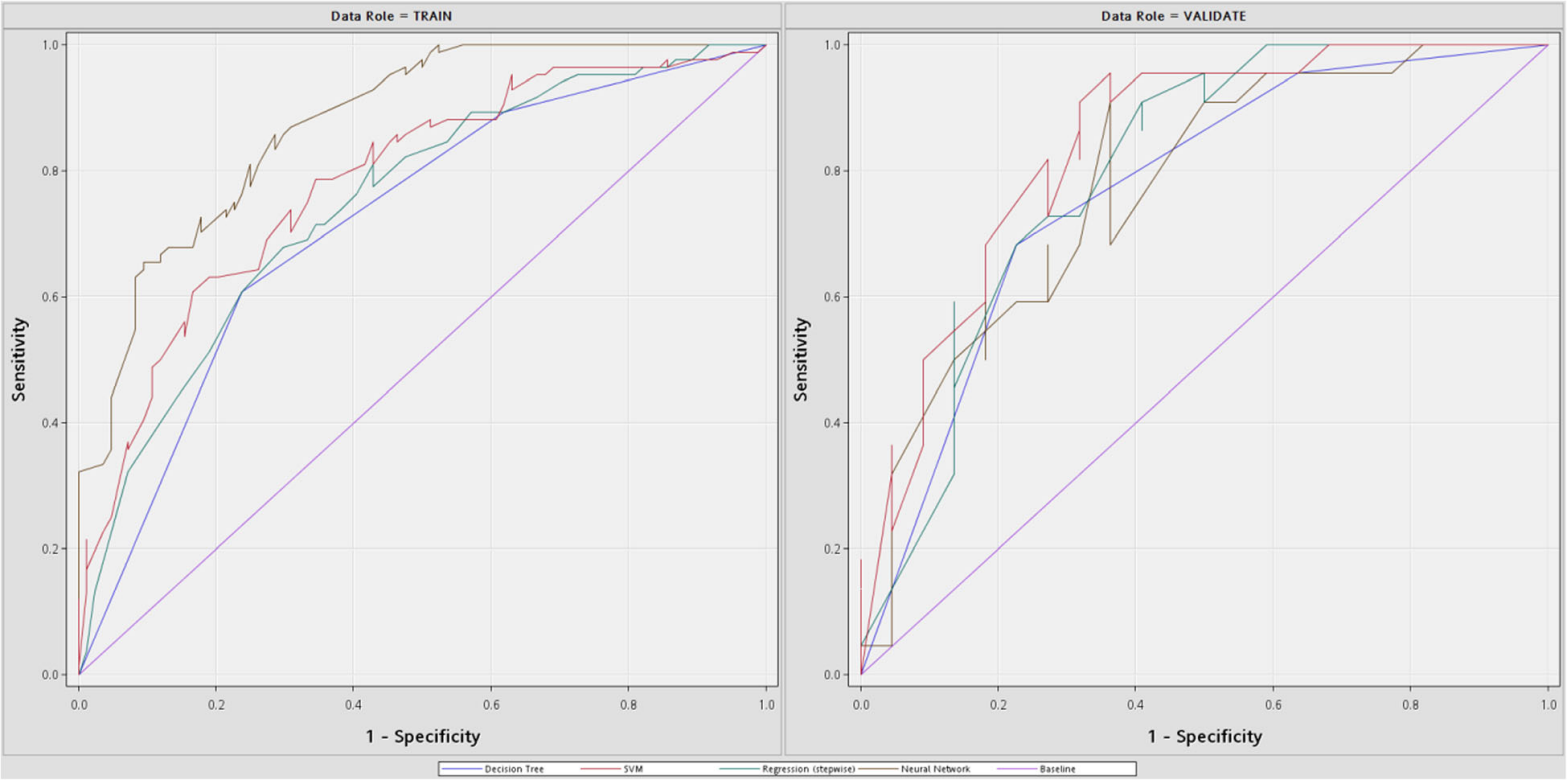
ROC plots for the training and test data sets. ROC curves of different machine learning algorithms (i.e., DT, LR, SVM, and ANN) for the training (80%) and test (20%) data sets

To compare with other studies, we incorporated percentage split in the first experiment and randomly divide our data set into 80% as training set and 20% as test set. We applied four machine learning algorithms to predict diabetic retinopathy, and the predictive performance ROC plots are shown in Table 3 and Fig. 1, respectively. The analyses of SVM, LR, ANN, and DT in Table 3 took 2.35, 1.41, 1.08, and 2.89 s, respectively, with SAS Enterprise Miner 13.1 software in HP z230 workstation with Intel Core i5–4690 3.5GHz and 8GB memory. In the test set, the AUC ranked from high to low were SVM, LR, ANN, and DT. Among the four machine learning algorithms, SVM classifier achieved the best prediction performance with 0.839, 0.795, and 0.933 in AUC, accuracy (Acc.), and sensitivity (Sens.), respectively, and ranked the second with 0.724 in specificity (Spec.). This indicates that advanced machine learning algorithms such as SVM and ANN perform better than the other classifiers for predicting diabetic retinopathy. In addition, it is observed that several machine learning algorithms (i.e., SVM, LR, and DT) achieved higher prediction performance in test sets, instead of training sets. This suggests that the prediction performance might be overestimated if the data set was merely divided into two data sets (i.e., as most previous studies used), and the test set is used for parameter tuning and model selection. Therefore, we incorporated a three-way data split scheme to prevent overestimation of predictive performance in the next section.

**Table 3:** Prediction performance using percentage split

Model	Training				Test			
	AUC	Acc.	Sens.	Spec.	AUC	Acc.	Sens.	Spec.
SVM	0.783	0.708	0.787	0.664	0.839	0.795	0.933	0.724
LR	0.749	0.679	0.703	0.660	0.802	0.727	0.813	0.679
ANN	0.875	0.762	0.756	0.768	0.777	0.682	0.682	0.682
DT	0.719	0.685	0.660	0.718	0.768	0.727	0.708	0.750

AUC, accuracy, sensitivity, and specificity of different machine learning algorithms using training (i.e., 80%) and test (i.e., 20%) data sets are evaluated

^a^Best evaluation measures in test set are underlined

In addition to percentage split, we also incorporated five-fold cross-validation to evaluate our method as shown in Table 4. Our data set was randomly divided into five folds, and each time one fold was regarded as the test set while the other four folds were used to train the prediction model. The above process was repeated five times until all folds took turns to serve as the test set. The evaluation measures obtained from these repetitions were averaged and listed in Table 4. It was also observed that SVM performed the best with 0.821, 0.791, 0.819, and 0.782 in AUC, accuracy, sensitivity, and specificity, respectively.

**Table 4:** Prediction performance using five-fold cross-validation

Model	Five-fold cross-validation			
	AUC	Acc.	Sens.	Spec.
SVM	0.821	0.791	0.819	0.782
LR	0.756	0.763	0.761	0.742
ANN	0.738	0.731	0.692	0.727
DT	0.690	0.718	0.683	0.729

AUC, Accuracy, sensitivity, and specificity of different machine learning algorithms using five-fold cross-validation are evaluated

### Prediction performance evaluated by three-way data split

**Fig. 2:**
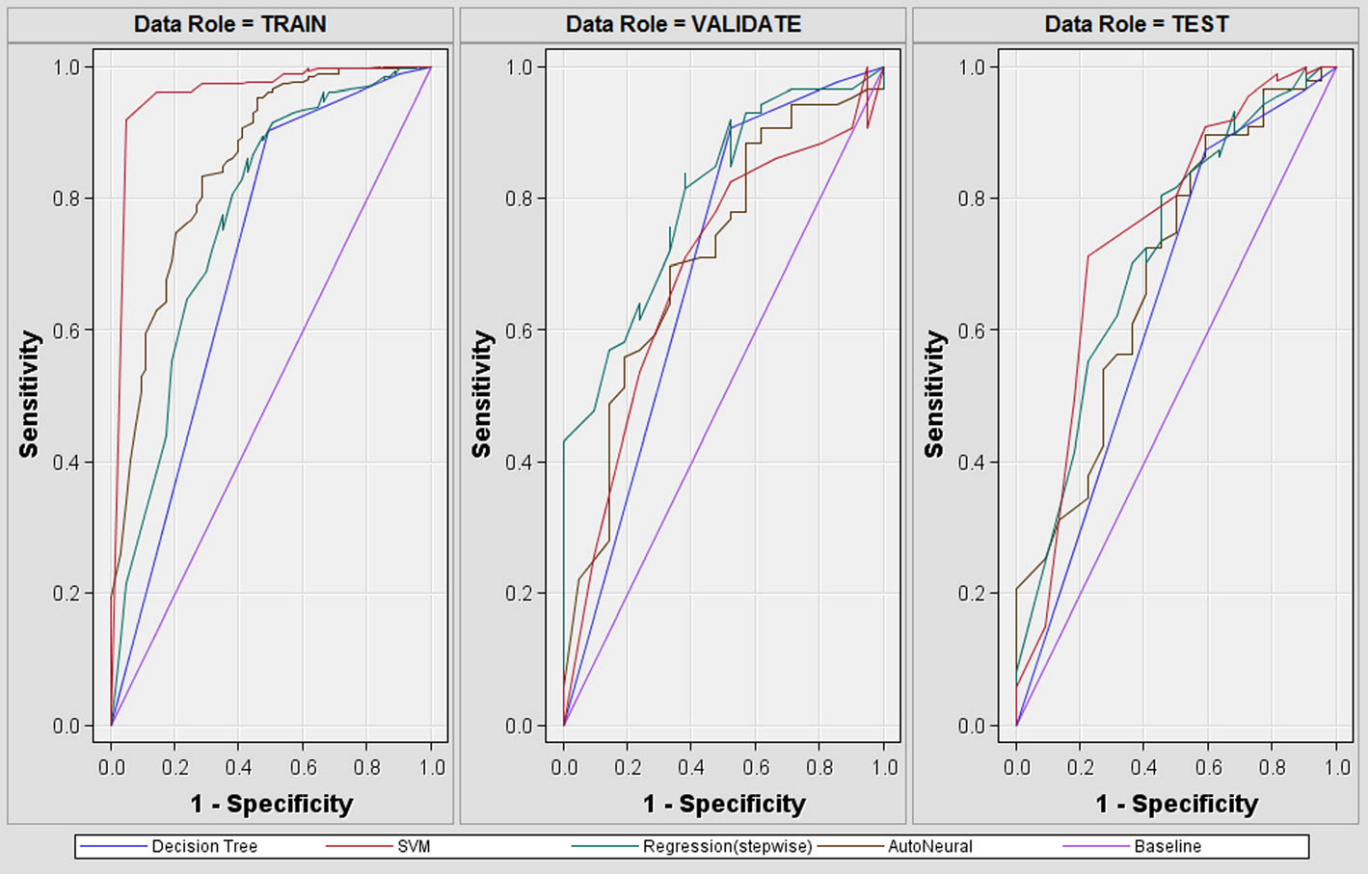
ROC plots for the training, validation, and test data sets. ROC curves of different machine learning algorithms (i.e., DT, LR, SVM, and ANN) for the training (60%), validation (20%), and test (20%) data sets

To avoid performance overfitting and evaluate real prediction performance, we further incorporated three-way data split in the second experiment and randomly divide our data set into 60% as training set, 20% as validation set, and 20% as test set. The prediction performance and ROC plots of three-way data split are demonstrated in Table 5 and Fig. 2, respectively. Compared with other machine learning algorithms, SVM achieved the highest prediction performance in terms of both accuracy and AUC in the validation set, which is often used for parameter tuning or model selection. These findings correspond well with our first experiment evaluated by percentage split. Therefore, this suggests that the proposed method to predict diabetic retinopathy is quite stable with respect to machine learning algorithms, and this also concludes that SVM classifier should be selected as the best model to predict diabetic retinopathy. For evaluation based on independent test set (i.e., as known as external validation), our SVM model achieved 0.817 in accuracy and 0.744 in AUC. In addition, when comparing Table 5 with Table 3, the prediction performance of test sets evaluated by three-way data split scheme are slightly lower than that by percentage-split. This implies that this observation that test accuracy is better than training accuracy could be resulted from overestimation. Therefore, this suggests that incorporation of three-way data split scheme is a better way to evaluate the real performance. However, to compare with other studies, we follow their percentage split evaluation and used prediction performance of SVM classifier in the test set of Table 3 for comparison in the next section.

**Table 5:** Prediction performance using three-way data split

	Training		Validation		Test	
Model	Acc.	AUC	Acc.	AUC	Acc.	AUC
SVM	0.863	0.961	0.822	0.801	**0.817**	**0.744**
LR	0.831	0.769	0.813	0.707	0.798	0.712
ANN	0.872	0.849	0.794	0.707	0.780	0.685
DT	0.825	0.707	0.817	0.693	0.780	0.640

Accuracy and AUC of different machine learning algorithms using training (i.e., 60%), validation (i.e., 20%), and test (i.e., 20%) data sets are evaluated

^a^Best evaluation measures in validation set are underlined as selected mode and independent performance evaluation is shown in bold

### Performance comparison with previous studies

Several previous studies have incorporated machine learning algorithms to predict diabetic retinopathy and the performance of their proposed approaches are summarized in Table 6. Hosseini et al. used logistic regression combined with backward elimination as feature selection to predict diabetic retinopathy from outpatient clinical data in Iran. Evaluated on the training set of 3734 patients (i.e., neither data partition nor cross-validation was used), they obtained AUC, sensitivity, and specificity as 0.704, 0.603, and 0.694, respectively. Oh et al. incorporated sparse learning models to analyze health records, including demographical data, medical history, blood tests, and urine tests, from the Korea National Health and Nutrition Examination Surveys (KNHANES) for diabetic retinopathy risk assessment in South Korea. They first collected a study population of 490 patients and randomly selected 67% of the population as training set (i.e., 327 patients) and the remainder as test set (i.e., 163 patients as internal validation group). Using least absolute shrinkage and selection operator (LASSO) combined with Bayesian information criterion (BIC) to evaluate internal validation group, they obtained the best AUC, accuracy, sensitivity, and specificity of 0.81, 0.736, 0.774, and 0.727, respectively [14]. Ogunyemi et al. applied ensemble classifiers to detect diabetic retinopathy from clinical data of 513 patients from urban safety net clinics as well as the public health data from the National Health and Nutrition Examination Surveys (NHANES) in the United States. Evaluated on the clinical data, classifiers were modestly predictive of retinopathy with the best model (i.e., RUSBoost ensemble classifier using only selected features on 20% set-aside test set) having AUC of 0.72, accuracy of 0.735, sensitivity of 0.692, and specificity of 0.559 [15].

**Table 6:** Performance of previous studies

Approaches	Data Sets	AUC	Acc.	Sens.	Spec.
Hosseini et al.	Iran	0.704	NA^a^	0.603	0.694
Oh et al.	South Korea	0.820	0.752	0.721	0.760
Ogunyemi et al.	United States	0.720	0.735	0.692	0.559

AUC, accuracy, sensitivity, and specificity of the best predictive performance reported in previous studies are summarized

^a^NA stands for “Not Available” because this evaluation measure was not reported in the study

We have tried our best to obtain the data sets from Iran, South Korea, and United States collected in the previous studies for performance comparison. Although the best way for comparison between our method and previous approaches is to run our proposed method on the data sets collected in previous studies, however, we did not succeed to obtain the data sets from publicly available databases nor email inquiries to the authors. Therefore, we ran the previously published methods on the Taiwan data set and compared with other approaches based on the same computational methods. The performance comparisons with Hosseini et al., Oh et al., and Ogunyemi et al. are illustrated in Table 7. For each comparison, we followed exactly the same experimental settings and incorporated identical machine learning algorithms proposed in these studies to show the performance of Taiwan data set. The numbers of patients and the numbers of features from different data sets are also summarized in Table 7. First, we used logistic regression combined with backward elimination to evaluate the Taiwan data set and obtained AUC, accuracy, sensitivity, and specificity of 0.796, 0.717, 0.745, and 0.689, respectively. Based on a similar number of features on a much smaller data set, we have achieved better performance compared to the Iran data set except for slightly lower specificity (as shown in comparison 1 of Table 7). Secondly, we incorporated LASSO combined with BIC on the Taiwan data set and achieved 0.823, 0.771, 0.784, and 0.757 in AUC, accuracy, sensitivity, and specificity, respectively (as shown in comparison 2 of Table 7). We also obtained slightly higher performance on the Taiwan data set in most measures except for specificity. Although the performance of two additional data sets (i.e., an external validation group of 562 patients and a newly-diagnosed group of 144 patients) were also reported in Oh et al., we compared our performance with the internal validation group since we do not have additional data sets and our data set was too small to be further divided into more data sets for external validation or independent test. Thirdly, we randomly selected 20% of data as test set and ran RUSBoost ensemble classifier on the training set. As shown in comparison 3 of Table 7, we obtained 0.744, 0.667, 0.682, and 0.650 in AUC, accuracy, sensitivity, and specificity, respectively. Compared to the data set from the United States, we achieved better specificity and AUC but obtained lower accuracy and sensitivity. In fact, Ogunyemi et al. applied RUSBoost to handle class imbalance problem and enhance prediction performance in their study. However, since our data set is more balanced and RUSBoost did not further improve performance (i.e., AUC = 0.744 and Acc. = 0.667) compared to logistic regression in comparison 1 (i.e., AUC = 0.796 and Acc. = 0.717) and LASSO in comparison 2 (i.e., AUC = 0.823 and Acc. = 0.771). This lends support to our assumption that appropriate machine learning algorithms combined with discriminative clinical features could effectively detect diabetic retinopathy, and thus increase cost-effectiveness in health care systems. Thus, as shown in Table 3, we incorporated the SVM model which achieved the best performance with 0.839 in AUC and 0.795 in accuracy as our proposed method to identify diabetic retinopathy.

**Table 7:** Performance comparison with previous studies

Approaches	Data Sets	Patients	Features	AUC	Acc.	Sens.	Spec.
*Comparison 1*							
Hosseini et al.	Taiwan	212	10	0.796	0.717	0.745	0.689
	Iran	3734	11	0.704	NA^b^	0.603	0.694
*Comparison 2*							
Oh et al.	Taiwan	212	10	0.823	0.771	0.784	0.757
	South Korea	490	37	0.820	0.752	0.721	0.760
*Comparison 3*							
Ogunyemi et al.	Taiwan	212	10	0.744	0.667	0.682	0.650
	United States	513	24	0.720	0.735	0.692	0.559

AUC, accuracy, sensitivity, and specificity of our Taiwan data set are compared with the Iran data set in Comparison 1 (i.e., using Hosseini et al.’s approach), with the South Korea data set in Comparison 2 (i.e., using Oh et al.’s approach), with the United States data set in Comparison 3 (i.e., using Ogunyemi et al.’s approach)

^a^Best evaluation measures in each comparison are underlined

^b^NA stands for “Not Available” because this evaluation measure was not reported in the study

## Discussion

### Clinical interpretation of selected features

**Fig. 3:**
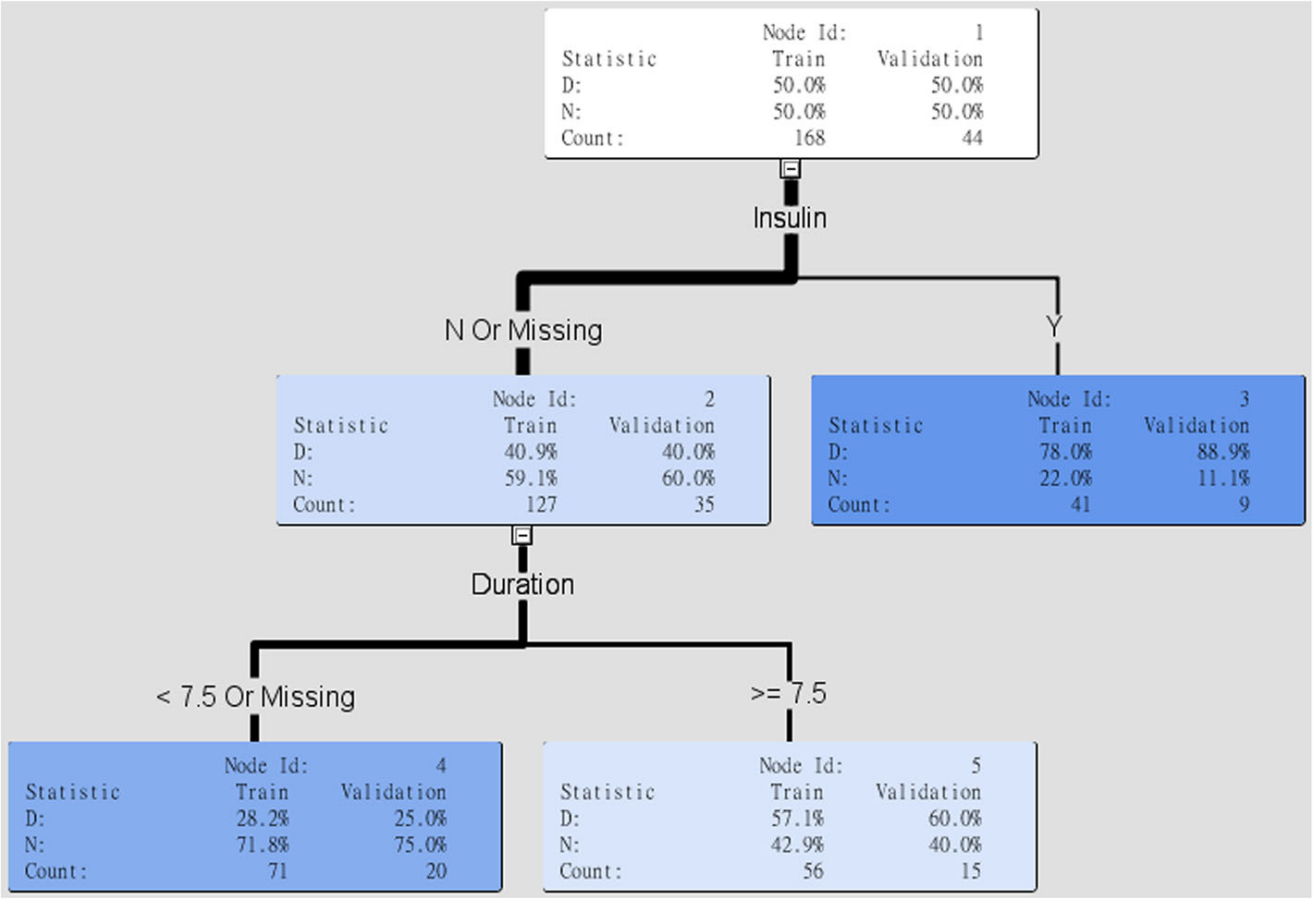
Clinical intepretation using decision trees. Interpretable rules for clinical practice generated by decision tress

The aim of this study is not only to achieve an accurate prediction performance, but also to generate an interpretable model for clinical practice. Figure 3 demonstrated the interpretable rules generated by decision tree models. In the decision tree model, insulin treatment was selected as the first variable to separate DR and normal patients. We discovered that in the insulin treatment group, the high DR development was 88.9%. Moreover, in the insulin absence group, the higher risk group was the patients with DM duration greater than or equal to 7.5 years (i.e., DR: 60%, Normal: 40%). When duration of DM is smaller than 7.5 years, less development of DR (i.e., DR: 25%, Normal: 75%). In summary, use of insulin and longer duration of DM were major predictors of DR in the decision tree models.

As for logistic regression models, we applied stepwise selection to identify important variables. The final logistic regression model consists of the following effects: duration and insulin. In the analysis of maximum likelihood estimates as shown in Table 8, duration and insulin therapy were also significant for DR. If duration of DM increases by 1 year, the odds ratio to have DMR is increased by 9.3%. The odds ratio to have DR is increased by 3.561 times for patients who use insulin compared to patients who do not use insulin.

**Table 8:** Odds ratio estimates of important risk factors

Effects		Point Estimates
Duration		1.093
Insulin	Y vs. N	3.561

Odds ratio estimates of duration and insulin variables generated by logistic regression model

### Effects of different years in duration of diabetes

If the features were only chosen from the already known risk factors, we might miss important unknown risk factors. However a variety of systemic and non-systemic features exist to inform possible risk factors of DR. Therefore our study decides to include all features from the database. We used data mining software to predict risk factors of DR, and all data mining test show the same result. Longer duration of diabetes and insulin therapy may predict diabetic retinopathy. Longer duration of diabetes is the major risk factor of DR and reviewed in many studies. Impaired glucose tolerance (IGT) precedes diabetes, if we track the time from the IGT to the diagnoses of diabetes, we can make sure the onset time of diabetes. Unfortunately, IGT is asymptomatic, and few studies monitor IGT.

The China Da Qing Diabetes Prevention Outcome Study (CDQDPOS) demonstrated rising cumulative incidence rate of severe retinopathy during 20-year follow-up for people with IGT [16]. It enhances our understanding of the development of microvascular complication such as nephropathy, neuropathy, and retinopathy. The CDQDPOS has suggested by tracking the duration of DM, it was found that the longer duration for DM, the higher incidence of retinopathy. Besides, good medical therapy for diabetes increase survival rate and may lead to longer life span and therefore the rate of developing retinopathy. In CDQDPOS, lower cumulative rate of retinopathy in the intensive group was achieved by life-style modification. Therefore, intensive glucose control, even though life style modification only can be a useful method to prevent from retinopathy.

Therefore, to investigate the effect of different years in duration of diabetes, we build decision trees with 2-year, 10-year, and 15-year duration of diabetes, regardless of insulin therapy, and the prediction performance is shown in Table 9. The duration of DM less than 2 years was negative predictor of DR with very low accuracy (i.e., 0.5). Accuracy from high to low was 10-year, 15-year, and 2-year. Accuracy was 0.705 based on the 10-year decision model tree. Both 10-year and 15-year tree favor higher duration could be the predictor of DR.

**Table 9:** Performance comparison of different years in duration

Model	Training Acc.	Test Acc.
DT (10-yr)	0.649	0.705
DT (15-yr)	0.601	0.659
DT (2-yr)	0.512	0.500

Ranked prediction performance of decision trees based on 2-year, 10-year, and 15-year duration of diabetes

### Findings and limitations of this study

There are many different risk factors in the worldwide. Although insulin therapy is not the traditional risk factor, there were many studies mentioned insulin therapy as a risk factor in some specific group. In the module of decision tree, we can try to build a clinical decision and provide the opinion for clinical decision making. Our data is not large; therefore data mining software can build a small tree easily. Decision trees from large data could be more complex, and difficult to make clinical decisions.

However, if one needs to search for highly reliable decision trees, our method can be used to select discriminative features for generating interpretable rules for clinical practice. The advantage of using decision trees is that cut-offs of variables can be manually specified in order to approximate an effective layer, and to elevate the levels of positive or negative correlations. We compare with multiple machine learning algorithms, including DT, SVM, ANN, and LR. SVM achieved the most accurate prediction performance.

The group we studied was based on a small population is local northern Taiwan. Duration of DM and insulin therapy was specific risk factors in the small group of diabetic patients. We only provide individual analysis for the small group; the result cannot be the risk factor for the population in Taiwan. The incidence of sight-threatening diabetic retinopathy (STDR) in Taiwan increased drastically in diabetic population during 2005–2011 [17]. However by observing separately for each gender, it was found that the incidence was lower in women, but higher in men. Furthermore, the age-adjusted prevalence rates of STDR decrease for both genders, which may be due to good monitoring strategies for caring diabetes, such as regular screening for retinopathy annually, and early therapy for retinopathy. Different incidence was observed in the different gender, we can analysis the difference or the DM in both gender.

## Conclusions

In this study, to predict diabetic retinopathy, we first extract demographical variables, laboratory test results, family history of diabetes, and exercise habits from patients. Then, we applied different machine learning algorithms to both achieve accurate prediction and identify novel risk factors. Experimental results demonstrate that support vector machines achieved the best performance with 79.5% and 0.839 in accuracy and AUC, respectively. Decision trees and logistic regressions both select use of insulin and duration of diabetes as the most discriminative features to predict diabetic retinopathy. Our results can be used to facilitate development of clinical decision support systems for clinical practice in the future.

## Methods

### Machine learning algorithms to predict diabetic retinopathy

DR prediction can be regarded as a multi-class classification problem. We incorporated decision trees, logistic regression, artificial neural networks, and support vector machines to predict DR. The SAS Enterprise Miner version 12.1 software of was used to generate prediction models.

To support decision making processes, we used decision trees to generate interpretable rules for clinical practice. We construct decision trees and generate rules for clinical decision making, categorizing based on data collection and categorical analysis, and generating decision trees as predicting models to assist with clinical decision making.

Logistic regression measures the relationship between the categorical dependent variable and one or more independent variables by estimating probabilities. The first assumes a logistic function and the second a standard normal distribution function. The odds of the dependent variable equaling a case are equivalent to the exponential function of the linear regression expression. This illustrates how the logic serves as a link function between the probability and the linear regression expression. We also incorporated stepwise selection to select discriminative features in logistic regression.

SVM classifier is a machine learning algorithm proposed by Vapnik based on structural risk minimization principle of statistics learning theory. It can be used to solve classification and regression problems. As prediction of diabetic retinopathy is a binary classification problem, SVM would be useful for our purpose. In the process of model development, we use radial basis function (RBF) as the kernel function in SVM.

Artificial neural networks are a family of statistical learning models inspired by biological neural networks and are used to estimate or approximate functions that can depend on a large number of inputs and are generally unknown. ANNs are generally presented as systems of interconnected neurons which send messages to each other. The connections have numeric weights that can be tuned based on experience, making neural nets adaptive to inputs and capable of learning.

### Experiment design and evaluation measures

For data partition, we followed percentage split approach from previous studies [15, 16] and randomly divide our data set as 80% training set and 20% test set. The training set is used to train a predictive model. The test set is incorporated to evaluate the real performance of a prediction method. To avoid performance overfitting, we also incorporated three-way data split to randomly divided our data set as 60% training set for model training, 20% validation set for model selection, and 20% test set for performance evaluation. As for the evaluation measures, we used accuracy, sensitivity, specificity, and area under the receiver operating characteristics (ROC) curve to compare with other previous studies. The accuracy (Acc.) of a prediction method is calculated as the summation of correct predictions divided by the total number of data, i.e. (*tp* + *tn*)/(*tp* + *fp* + *tn* + *fn*) where *tp*, *fp*, *tn*, and *fn* represents true positives, false positives, true negatives, and false negatives. Sensitivity (Sens.) and specificity (Spec.) are defined as *tp*/(*tp* + *fn*) and *tn*/(*tn* + *fp*), respectively. The accuracy was also used for model selection in our experiment. The area under the ROC curve (AUC) is used to assess performance during parameter selection, and is one of the most appropriate measures of performance as it is non-parametric and threshold independent. In an ROC curve, the true positive rate (i.e., sensitivity) is plotted in function of the false positive rate (i.e., 1-specificity) for different cutoff points of a parameter.

## Abbreviations

ANN: Artificial neural networks
AUC: Area under the ROC curve
BMI: Body mass index
DPB: Diastolic blood pressure
DR: Diabetic retinopathy
DT: Decision trees
HbA1C: Hemoglobin A1C
IGT: Impaired glucose tolerance
LR: Logistic regression
NPDR: Non-proliferative diabetic retinopathy
PDR: Proliferative diabetic retinopathy
ROC: Receiver operating characteristic
SBP: Systolic blood pressure
SMBG: Self-monitoring blood glucose
STDR: Sight-threatening diabetic retinopathy
SVM: Support vector machines
T2D: Type 2 diabetes
